# A Novel Wearable Device for Continuous Temperature Monitoring & Fever Detection

**DOI:** 10.1109/JTEHM.2021.3098127

**Published:** 2021-07-19

**Authors:** Nishant Verma, Iman Haji-Abolhassani, Suhas Ganesh, Jesus Vera-Aguilera, Jonas Paludo, Roxana Heitz, Svetomir N. Markovic, Kimary Kulig, Atiyeh Ghoreyshi

**Affiliations:** 1 Verily Life Sciences South San Francisco CA 94080 USA; 2 Division of HematologyMayo Clinic Rochester MN 55905 USA; 3 Division of Medical OncologyMayo Clinic Rochester MN 55905 USA

**Keywords:** Continuous temperature monitoring, wearable devices, febrile neutropenia, early fever detection, machine learning

## Abstract

*Objective:* Continuous temperature monitoring in high-risk patients can enable healthcare providers to remotely track patients’ temperatures, promptly detect fevers and timely intervene to improve clinical outcomes. We evaluated if a novel wearable, continuous temperature monitor (Verily Patch) can reliably estimate body temperature and early detect fevers in an outpatient setting in patients at a high risk of febrile neutropenia (FN) who recently underwent chemotherapy and autologous stem cell transplantation (ASCT). *Methods:* 86 patients at a high risk for FN were prospectively enrolled at Mayo Clinic, MN. Patients wore the device in their axilla region for 7 days post ASCT and recorded self-measured oral temperatures every 3 hours. Patients were also followed using clinical standard-of-care procedures with daily oral temperature assessment. The clinic- and patient-assessed oral temperatures were used to develop and evaluate Verily Patch’s body temperature and early fever detection algorithms using a K-fold cross-validation approach. *Results:* The Verily Patch reliably measured body temperatures with an error of 0.35 ± 0.88°F in comparison to clinic- and patient-assessed oral temperatures. The sensitivity and specificity of the patch in detecting clinic-assessed fever episodes was 90.2% and 87.8%. The patch detected 14.3 times the number of clinic-assessed fever episodes with a median lead time of 4.3 hours. *Conclusion:* Patient self-monitoring of temperature and fever incidents suffers from low accuracy and is impractical for extended periods of time. Continuous temperature monitoring by a wearable device (such as Verily Patch) has the potential to overcome these challenges resulting in better patient clinical outcomes and more cost-effective care.

***Clinical and Translational Impact Statement—*** Verily Patch can enable healthcare professionals to remotely track patients’ body temperatures and promptly intervene at the first signs of fever, thereby improving patient outcomes and reducing healthcare costs.

## I. Introduction

The current clinical standard-of-care method for monitoring a patient’s body temperature is to periodically take a measurement every few hours. In an outpatient setting, the frequency of temperature measurements is typically even more infrequent. This results in a delay in fever detection and subsequent treatment adversely affecting patient outcomes, especially in high risk populations. One such population is cancer patients who have undergone high-dose chemotherapy and are at a high risk for febrile neutropenia (FN). FN is often the first and the only sign of infection following high-dose chemotherapy [1]. FN is a medical emergency. An estimated 4,000 people die of FN each year in the United States, with this number expected to rise due to the increasing incidence of cancer [2], [3]. A study published in 2012 estimated total costs of $\$ $2.3 billion for adults and $\$ $439 million for children for cancer-related neutropenia hospitalizations in the U.S. [4].

A particularly high risk group for FN are patients undergoing stem cell transplantation (SCT). These patients frequently experience prolonged neutropenia that may last 14 days or more. It is estimated that more than 80% of patients undergoing SCT will experience at least one episode of FN [1]. FN is defined as a one-time oral temperature of greater than 38.3° (approximately 100.9°F) or a sustained temperature of greater than 38° (100.4°F) for ≥1 hour in a patient who has an absolute neutrophil count (ANC) of less than 500 cells/$\mu \text{L}$ [5]. All patients with FN are treated empirically with broad-spectrum antibiotics promptly at the first sign of infection or fever. Prompt identification and treatment of FN is imperative as delays in antibiotic treatment is associated with a significant mortality risk due to serious infection [1], [5]–[7].

However, screening for FN is challenging due to a lack of consensus on temperature measurements and thermometers. While some devices are invasive and poorly tolerated, others require healthcare professional support resulting in infrequent temperature monitoring [2]. Patient self-monitoring of temperatures at home is sometimes recommended. However, it is impractical for prolonged periods of monitoring. Low-cost, widely available, non-invasive and continuous monitoring devices for early identification of fever in high-risk populations have the potential to improve patient outcomes and decrease healthcare costs.

In this study, we evaluated a low-cost, novel wearable, continuous temperature monitoring device (Verily Patch, Verily Life Sciences) in its ability to measure body temperature and early detect fevers in an outpatient setting in patients undergoing ASCT for hematological malignancies. We compared the device’s performance with the clinical standard-of-care monitoring and patient self-monitoring of temperatures in an at-home setting.

## II. Methods

### A. Data Sources & Study Population

**FIGURE 1.**
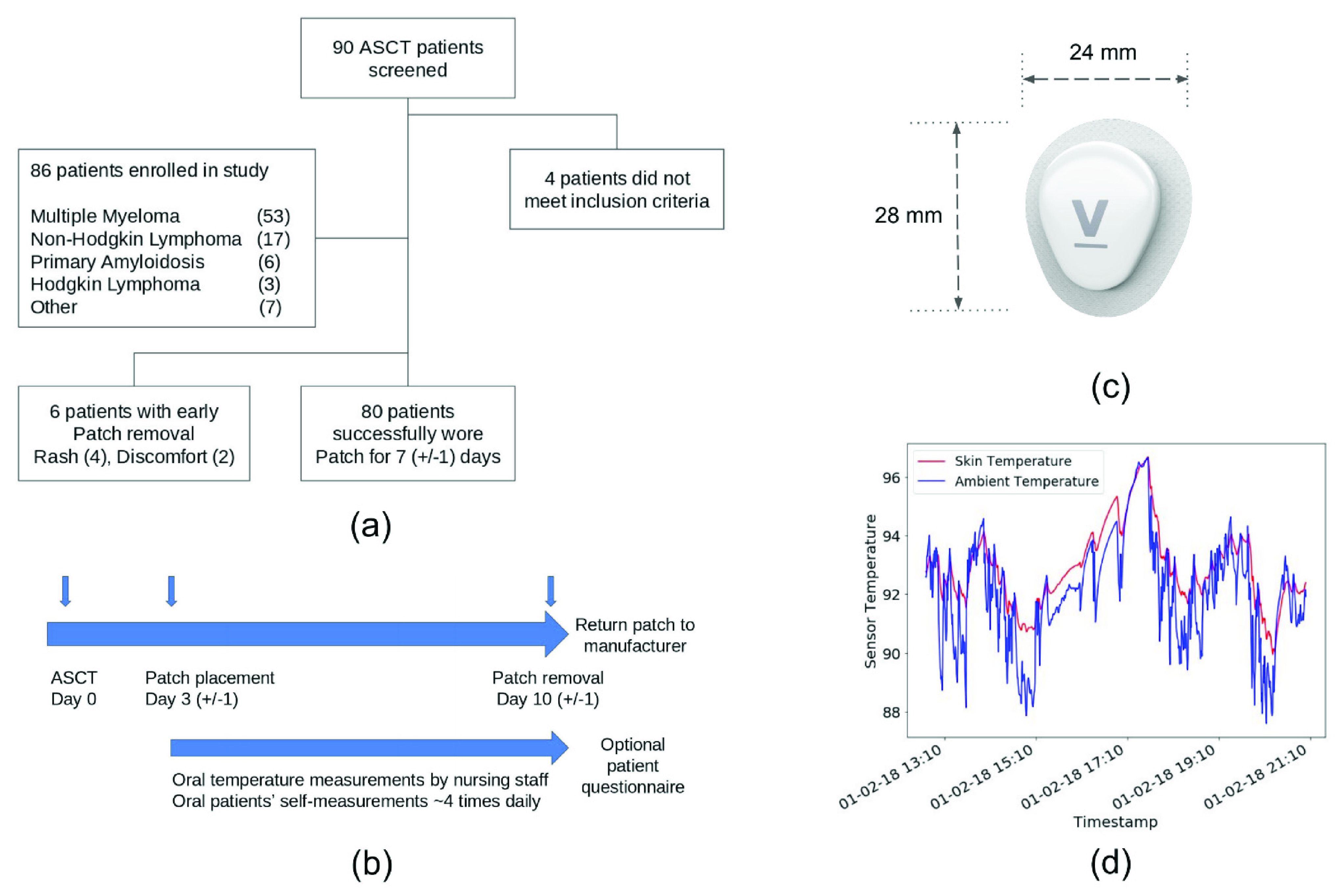
Schematic illustration showing (a) patient enrollment and characteristics, (b) study timeline showing device application, removal and clinic- and patient-assessed oral temperature measurements, (c) Verily Patch device, and (d) a sample signal from device’s skin and ambient sensors.

Adult patients (n = 90) diagnosed with hematologic malignancies undergoing high-dose chemotherapy followed by autologous stem cell transplantation (ASCT) at Mayo Clinic, MN were screened for this study. We prospectively enrolled 86 patients between June 2018 and March 2019 on a study protocol approved by the Mayo Clinic Institutional Review Board (Figure 1a). Informed consents for data collection were obtained from the patients and data was de-identified by Mayo Clinic before sharing with Verily Life Sciences.

On day 3 (±1) post-ASCT, research staff activated and placed the Verily Patch device in patients’ axilla regions. The skin was prepped before the patch placement in the same manner according to Mayo’s standard operating procedure for central line placement. The wearable device was placed on the opposite side of the central line location (i.e. port, PICC, IVAD, etc.) when existent. Patients were given an instruction card to take home explaining the patch device, study activities and any special care instructions. The patch was worn continually and removed 7 (±1) days after placement. Patients with a known allergy to adhesives or bandages were excluded from this study. Patients were instructed to immediately remove the patch in case of any adverse skin reactions (such as irritation) or localized device heating and contact the study staff. Patients were followed daily as outpatients at the Mayo Clinic Hematology/Bone Marrow Transplant Unit until neutrophil engraftment (defined as 3 consecutive days with an ANC of $0.5\times 10 ^{9}$/L or 1 day with a count of $1.0\times 10 ^{9}$/L). During these visits, the clinical staff measured patient vital signs including body temperature using Mayo Clinic’s standard-of-care monitoring tools (SureSigns VS3 Vital, Phillips, accuracy: ±0.36°F). The day and time of these “clinic-assessed” oral temperatures were collected and recorded in an encrypted database for subsequent analysis. During these daily visits, the clinical staff also checked patients’ axilla regions where patch was applied for any symptoms like skin irritation or rash.

Additionally, starting at day 3 (±1) post-ASCT, patients were asked to complete oral temperature self-measurements throughout the study every 3 hours while awake (minimally 4 times per day) and at any additional time if there was a concern for fever. A commercially available digital thermometer (Iproven/DT-R1221A, accuracy: ±0.18°F) was given to the patients by the study staff who provided usage instructions. “Patient-assessed” oral temperatures were recorded by the patients in paper logs provided by the study staff. At the end of the study, the patch was returned to the manufacturer (Verily Life Sciences) for temperature data abstraction. An optional questionnaire was given to patients at the end-of-study to assess comfort and ease of use of the Verily Patch device. A summary of study timeline is shown in Figure 1b. Both patients and study personnel were blinded to the patch temperature reads.

### B. Verily Patch

Verily Patch is a custom designed, small form-factor (28mm $\times24$mm $\times7$mm), long lasting (3 months battery life), wearable adhesive patch for continuous temperature monitoring (Figure 1c). The device is worn in the axilla region and gently secured using a medical grade adhesive liner (tested per ISO 10993 standard for biocompatibility [8]). The device is waterproof (IPX7 rated) and designed for continuous usage by adults while participating in regular daily activities. The device utilizes two contact integrated circuit temperature sensors to continually sense skin and ambient temperatures every 30 seconds (Figure 1d). The temperature sensors are thermally isolated from heat generating electrical components and meet ASTM E1112-00 (2018) standard and measure temperature with an accuracy of 0 ± 0.36°F over the range of 14°F to 185°F.

In this study, the Patch’s temperature sensor data was stored on-device in an internal flash memory. Once the devices were returned to the manufacturer, the sensor data was downloaded wirelessly (using Bluetooth Low Energy) to a host computer for processing and analysis.

### C. Body Temperature Measurement Algorithm

Traditional axillary thermometers require armpits to be closed in order to reliably measure body temperature without getting affected by the ambient temperature. However, such an approach is impractical for continuous temperature monitoring. The Verily Patch uses a combination of skin and ambient temperature sensor measurements to reliably measure the body temperature. The continuous monitoring feature allows for the use of temporal dynamics across both the sensors as additional model inputs for body temperature estimation. An all-zeros Nonlinear AutoRegressive model with eXogenous inputs (NARX) model is used for estimating the body temperature ($T_{b}(t))$:

\begin{align*}&\hspace {-1pc} T_{b} (t) \\=&c+\sum \limits _{m=0}^{M} {\sum \limits _{p=1}^{P} {(w_{m,p}^{a} T_{a}^{p} (t-m)+w_{m,p}^{s} T_{s}^{p} (t-m)}} \\&+ w_{m,p}^{a,s} T_{a}^{p} (t-m)\times T_{s}^{p} (t-m))\tag{1}\end{align*}

where, c is an intercept term, ${\text{T}_{a}}$(t), ${\text{T}_{s}}$(t) represent the ambient and skin sensor measurements at time t, and w represent regression weights to be estimated. The regressors include the current sensor measurements (m = 0, p = 1), past sensor measurements (m = 1,…, M), higher degree polynomial terms (p = 2,…, P) of current and past sensor measurements, and interaction terms between skin and ambient sensor measurements. The regressors were normalized to zero mean and unit standard deviation and model was fitted with a ridge (L_2_ norm) regularization term to reduce overfitting.

This model was trained using both patient- and clinic-assessed oral temperature measurements as ground truth. Oral temperatures below 97°F (6%) were excluded from model training as outliers. Samples were appropriately weighted to correct any data imbalance and ensure a good model fit across the entire temperature range. The NARX model was trained and evaluated using a nested 10-fold cross-validation (CV) approach. An outer 10-fold CV layer was used to train the NARX model and assess its generalization performance. An inner 10-fold CV layer performed model selection by optimizing values of M, P, and ridge regularization parameter ($\alpha$). A maximum M = 5 number of past sensor measurements and a maximum polynomial degree of P = 5 were considered. Model selection was performed using the 1 standard error rule, where the most parsimonious model was chosen whose root mean squared error (RMSE) was within one standard error of the best model’s RMSE error. Since there are multiple measurements per patient, splitting in both CV layers was done at the patient-level to ensure all measurements from a given patient were either in the training set or the test set for a given CV split.

Body temperature measurements were obtained from the trained models ${\mathcal{ M}}_{\mathrm {i}},\,\,i = 1,\ldots $,K of outer CV and evaluated against patient- and clinic-assessed oral temperature measurements. Temperature measurement error was calculated as mean and standard deviation of differences between patch-measured body temperatures and oral temperatures.

### D. Fever Episode Detection Algorithm

We used the patch temperature measurements to develop a fever episode detection algorithm and evaluated its performance in early detection of fevers. We first evaluated the patch’s performance in static fever detection, defined as the ability to detect fever based only on the current temperature. A receiver operating characteristic (ROC) curve of the patch’s performance in static fever detection was generated using fever ground truths as clinic- and patient-assessed oral temperatures ≥ 100.4°F. A patch temperature threshold for static fever detection was determined as the operating point on the ROC curve closest to 100% sensitivity and 100% specificity. This threshold was used to detect whether a patient’s body temperature indicated fever or not at a given time point.

Static fever detections were post-processed to obtain more clinically meaningful fever episodes, defined as fever detections sustained over a time period. A patch fever “high confidence index” was calculated at each patch reading by assessing if the percentage of fevers detected in the past one hour crossed a defined “percentage threshold”. The percentage threshold is tunable depending on the desired sensitivity and specificity of fever episode detection. A fever episode started when the high confidence index went from low to high and ended when it went back to low. A smoothing layer was added to remove spurious predicted fever episodes and merge the nearby disconnected ones. This was achieved using a hysteresis filter as follows: the start of a fever episode is evoked once an unfiltered fever episode persists for more than 10 minutes and the fever episode ends when there is no unfiltered fever episode for more than 60 minutes.

We evaluated the sensitivity and specificity of the patch in early detection of fevers for different percentage thresholds of the high confidence index. The patch’s lead time in predicting clinic- and patient-assessed fevers was calculated as the time interval between the start of the fever episode measured by the patch’s smoothed algorithm, as defined above, and the time at which a fever was recorded by the clinic or by the patient. To avoid acquiring more than one lead time measure for any single fever episode prediction, if multiple clinic- or patient- recorded fevers were present for the same fever episode, only the earliest recorded fever was used in the lead time calculation.

### E. Analysis Software

All data analysis was performed in Python v3.6. Following libraries were used to implement the methods: (i) scikit-learn v0.24.0 for NARX algorithm, fever detection algorithm and cross-validation experiments, (ii) pandas v1.1.5, numpy v1.16.4 and scipy v1.2.1 for data analysis and signal processing, (iii) statsmodels v0.12.2 for statistical analysis, and (iv) matplotlib v3.3.4 for data visualization.

## III. Results

### A. Patient Characteristics

Of 90 patients screened for enrollment, 86 met inclusion criteria and were enrolled. The mean patient age was 58.7 (range 26-70 years old), 82.6% were male (n = 71) and 17.4% (n = 15) were female. The most common indication for ASCT was multiple myeloma (n = 53) followed by non-Hodgkin lymphoma (n = 17), amyloid light-chain amyloidosis (n = 6), Hodgkin lymphoma (n = 3), or other (n = 7). The patch was successfully worn continuously for 7 days by 93% of the patients (n = 80); 6 patients reported side effects of rash (n = 4) and discomfort (n = 2), these side effects led to early removal of the patch. In total, more than 13,000 hours of patch wear among 86 patients resulted in ~1.6 million data points. Clinic-assessed oral temperatures were recorded daily for every patient, resulting in 647 data points. Patient-assessed oral temperatures were recorded on an average every 4.4 hours resulting in a total of 2133 data points.

Of 65 patients voluntarily completing an end-of-study questionnaire, 89.3% found study participation “quite” or “somewhat” worthwhile with only one patient responding, “not at all”. Regarding comfort of wearing the patch, 95% reported it as “quite” or “somewhat” comfortable with only one patient responding, “not at all”. There was no difficulty in using the patch reported by 94% of responding patients, with the remainder reporting “somewhat” or “a little bit” of difficulty.

### B. Body Temperature Measurement Performance

**FIGURE 2.**
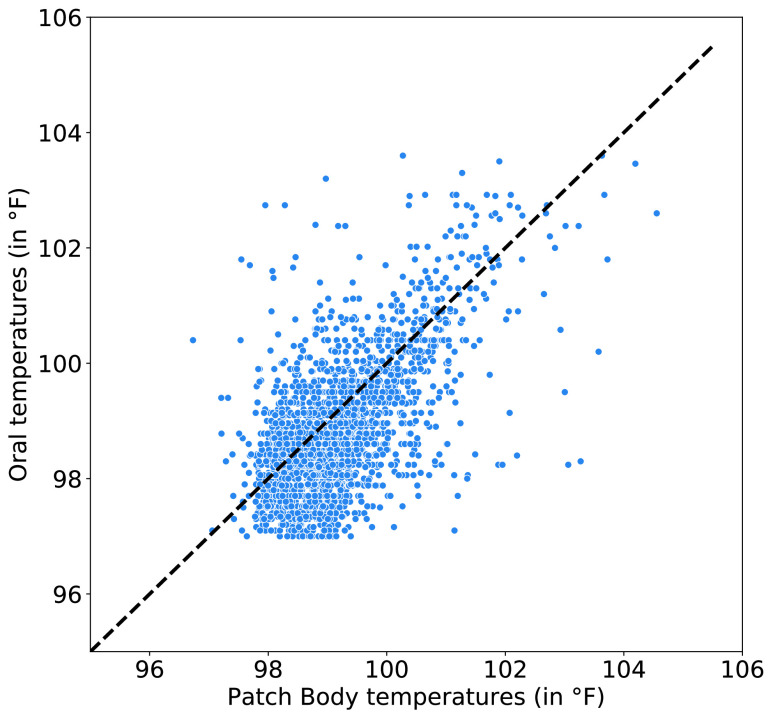
Agreement between clinic- and patient-assessed oral temperatures and patch measured body temperatures.

The inner CV consistently selected $M = 1$ past sensor measurement and a polynomial of degree $P = 4$ as the best parsimonious model. The ridge regularization parameter ($\alpha$) ranged between [10^−3^, 10^−2^] across the $K =10$ CV splits. When compared to all oral temperature readings (N = 2780), the patch measured body temperatures with an error of 0.35 ± 0.88°F (Figure 2). When compared to clinic-assessed (N = 647) and patient-assessed (N = 2133) oral temperatures separately, the patch measured body temperatures were much closer to clinic-assessed oral temperatures (error of 0.29 ± 0.77°F) than patient-assessed oral temperatures (error of 0.37 ± 0.90°F).

### C. Fever Episode Detection Performance

**FIGURE 3.**
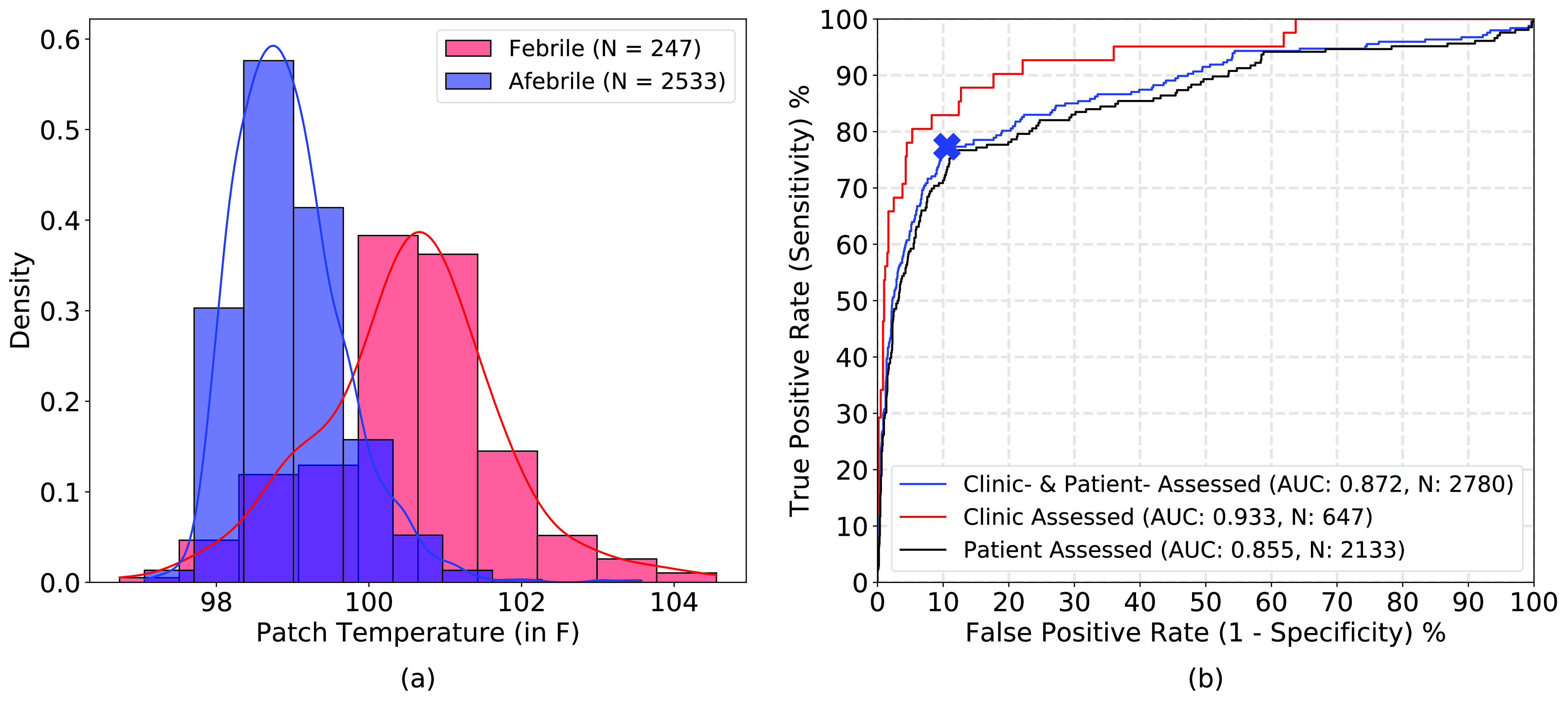
Static fever detection: (a) Distribution of patch measured body temperatures in febrile and afebrile groups, and (b) ROC curve of patch’s performance in static fever detection and best operating point (threshold: 99.83°F) shown as a blue cross. The area under the ROC curve (AUC) values are shown in the legend.

The patch temperature threshold for static fever threshold was found to be 99.83°F corresponding to the best operating point on the ROC curve (Figure 3). The static fever detection performance of the patch was significantly better for clinic-assessed fevers (AUC: 0.933) as compared to patient-assessed fevers (AUC: 0.855).

**FIGURE 4.**
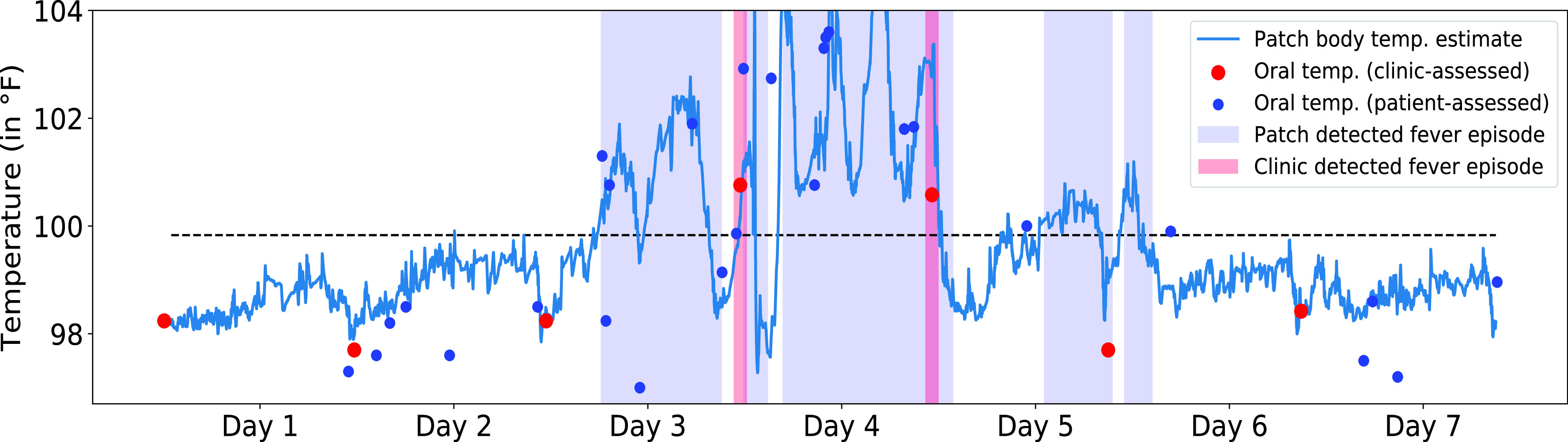
A representative case of the patch’s early fever episode detection versus clinic-recorded fevers. The plot also shows clinic- and patient-assessed oral temperatures.

Representative patch-predicted fever episodes for a single patient are shown in Figure 4 along with clinic- and patient-assessed oral temperatures. Table 2 shows sensitivity and specificity of the patch in early detection of clinic-recorded fevers for different high confidence index thresholds. Using the 50% high confidence index threshold, the patch is able to early detect clinic-recorded fevers with a sensitivity of 90.2% and specificity of 87.8%. In contrast, fever detection by patient self-assessed oral temperatures at a time point nearest (within 1 hour) to the clinic fever assessment resulted in 62% sensitivity and 93% specificity. The median lead time of patch’s prediction of clinic-recorded fevers was 4.3 hours (range of 0.2 – 18.4 hours) and the patch predicted 14.3 times the number of fever episodes vs. all clinic readings. Notably, the patch predicted one or more fever episodes during 39.8% of the time intervals that patients spent between clinic visits. Using the 50% high confidence index, the patch also detected patient-recorded fevers with a sensitivity of 72.5%, specificity of 84.3%, and a median lead time of 2.3 hours (range of 0 - 14.1 hours). The percentage threshold used for determining fever episodes can be tuned to achieve desired sensitivity, specificity, and lead times of fever detection (Table 2).

**TABLE 1:** Nested K-Fold CV for NARX Training & Evaluation

Require: $D$, dataset with regressors ($T_{a}$, $T_{s}$) and output $T_{b}$
Require: ${\mathcal{ M}}$, NARX model as defined in equation (1)
Require: Hyperparameters: $P$ (number of polynomial degrees), $M$ (number of past sensor measurements), $\alpha $ (ridge regularization parameter)
For $i = 1$ to $K$ splits do
Split $D$ into $D_{i}^{train} $ and $D_{i}^{test} $for the $i^{th}$ split
For $j=1$ to $K$ splits do
Split $D_{i}^{train} $ into $D_{j}^{train} $ and $D_{j}^{test} $for the $j^{th}$ split
For all $P =1$,…,5, $M =0$,…,5, $\alpha =0$, …, $A$ do
Train ${\mathcal{ M}}$ on $D_{j}^{train} $ with parameters set to $P$, $M$, $\alpha $
Calculate $RMSE_{P,M,\alpha }^{j} $on $D_{j}^{test} $
End for
End for
Calculate inner CV test error for all hyperparameter settings: $RMSE_{P,M,\alpha }^{cv} =\frac {1}{K}\sum \nolimits _{j=1}^{K} {RMSE_{P,M,\alpha }^{j}} $
Select optimal $P^{i}$, $M^{i}$, $\alpha ^{i}$ using 1 standard error rule
Train ${\mathcal{ M}}_{\mathrm {i}}$ on $D_{i}^{train} $with selected hyperparameters ($P^{i}$, $M^{i}$, $\alpha ^{i}$)
Calculate temperature predictions and ${RMSE}_{i} \text{ on }D_{i}^{test} $
End for
Calculate outer CV test error: $RMSE^{cv} =\frac {1}{K}\sum \nolimits _{i=1}^{K} {RMSE_{i}} $

**TABLE 2:** Tunability of Patch’s Fever Episode Detection: High Confidence Index Can be Tuned to Achieve Desired Level of Sensitivity, Specificity and Lead Times in Detecting Clinic-Assessed Fevers

High Confidence Index	Sensitivity (%)	Specificity (%)	Ratio of patch-predicted fever episodes to clinic-recorded fevers	Median fever prediction lead time to clinic-recorded fevers (hours)
0	95.1	76.4	23.0	6.2
20	90.2	85.3	15.5	5.3
40	90.2	87.6	14.4	4.8
50	90.2	87.8	14.3	4.3
60	87.8	88.7	14.2	3.5
80	78.0	91.4	14.5	3.3
100	70.7	94.2	13.7	2.6

## IV. Conclusion

Early detection of elevated temperatures in high-risk patients is critical for timely intervention and preventing any deterioration in clinical outcomes. Continuous temperature monitoring devices can enable health care professionals to continually track a patient’s temperature in both clinical and at-home settings. In this study, we evaluated the use of a new wearable continuous temperature monitor (Verily Patch) in a high-risk population of cancer patients who have recently undergone high-dose chemotherapy and stem cell transplantation. Neutropenia is a common complication of cytotoxic cancer therapy and associated with high risk for infections which are a major cause of morbidity, mortality and significant increase in the cost of care [9], [10]. Studies have shown that delayed administration of appropriate antibiotics adversely affects mortality rates [11], [12]. Therefore, early detection of fevers in this patient population is critical for achieving good clinical outcomes.

The clinical standard-of-care temperature monitoring is infrequent (ranging from hours to days), manual and requires the support of healthcare professionals. Patients at a high risk of FN are asked to self-monitor their temperature between clinical assessments and promptly report any fever incidences to medical staff. Since our goal is to evaluate the benefit of continuous temperature monitoring over the current standard-of-care, both clinic- and patient-assessed oral temperatures are considered in this study. Verily Patch measured body temperatures with a measurement error of 0.35 ± 0.88°F, which is comparable to accuracy of other non-invasive thermometers [13]–[16]. The measurement error was significantly higher for patient-assessed oral temperatures as compared to clinic-assess oral temperatures. This is expected because clinical thermometers are typically more accurate than commercial off-the-shelf thermometers used for self-monitoring. Moreover, self-assessed oral temperatures are susceptible to user errors (such as incorrect thermometer tip placement), which results in self-assessed oral temperatures being much noisier than clinic-assessed temperatures.

The imprecision of self-assessed oral temperatures is also evident in low sensitivity (62% sensitivity and 93% specificity) of self-assessed oral temperatures in predicting clinic-assessed fevers. In comparison, the patch detected clinic-assessed fevers with a high sensitivity and specificity of 90.2% and 87.8%, respectively. The patch predicted 14.3 times the number of fevers vs. all clinic readings with a median lead time of 4.3 hours. Notably, the patch picked up fevers in a large proportion of the time interval (39.8%) that patients spent between clinic appointments. This indicates that the patch has the potential to improve patient outcomes and reduce healthcare costs by early detection of fever and timely intervention before symptoms occur in patients. It is important to highlight that, while early fever detection can be a powerful tool, it should be coupled with a healthcare provider assessment to minimize false positive results and misuse of antimicrobial therapy.

This study was conducted to collect data needed to develop the Verily Patch algorithms for body temperature measurement and fever episode detection, and assess its potential in early fever detection. As such, this was a non-interventional study with both patients and clinic staff blinded to any patch temperature data. This study design has the limitations of not collecting information on infection diagnosis, antimicrobial administration and its timing, patient hospitalization duration and costs of intervention. This limited our ability to assess direct impact of Verily Patch on patient outcomes and healthcare costs. The accuracy of the Verily Patch in body temperature measurement and early fever detection is promising and suitable for a follow-on, randomized interventional study in a clinical setting. Future studies would benefit from collecting other vital signs such as heart rate and blood pressure to include along with continuous temperature monitoring for more accurate fever detection. In this study, research staff placed the devices in patients’ axilla regions and, therefore, placement locations are expected to be somewhat consistent. As part of a future study, a detailed investigation of the impact of patch’s placement location on reported body temperature should be conducted.

These preliminary results demonstrate that a continuously wearable dermal temperature sensor allowed accurate and early fever episode detection in patients with high likelihood of febrile neutropenia. These results could have significant clinical implications for cancer patients in the form of accurate and real-time fever detection, more rapid diagnosis, and earlier intervention which could potentially result in more cost-effective care with better patient clinical outcomes.

## References

[1] A. J. Zimmer and A. G. Freifeld, “Optimal management of neutropenic fever in patients with cancer,” J. Oncol. Pract., vol. 15, no. 1, pp. 19–24, Jan. 2019. 30629902 10.1200/JOP.18.00269

[2] K. Wang, “Non-contact infrared thermometers for measuring temperature in children: Primary care diagnostic technology update,” Brit. J. Gen. Pract., vol. 64, no. 627, pp. e681–e683, Oct. 2014. 25267058 10.3399/bjgp14X682045 PMC4173735

[3] C. Shah, X. Du, R. Bishnoi, and J. Bian, “Risk of mortality in adult cancer febrile neutropenia patients with a machine learning approach,” J. Clin. Oncol., vol. 36, no. 15, 5 2018, Art. no. e13562.

[4] E. Tai, G. P. Guy, A. Dunbar, and L. C. Richardson, “Cost of cancer-related neutropenia or fever hospitalizations, United States, 2012,” J. Oncol. Pract., vol. 13, no. 6, pp. e552–e561, Jun. 2017. 28437150 10.1200/JOP.2016.019588 PMC5470648

[5] Cost of Cancer-Related Neutropenia or Fever Hospitalizations, CDC, Atlanta, GA, USA, 2012. 10.1200/JOP.2016.019588 PMC5470648 28437150

[6] L. R. Baden, “Prevention and treatment of cancer-related infections,” J. Natl. Compr. Canc. Netw., vol. 10, no. 11, pp. 1412–1445, 2012. 23138169 10.6004/jnccn.2012.0146

[7] L. Nesher and K. V. I. Rolston, “The current spectrum of infection in cancer patients with chemotherapy related neutropenia,” Infection, vol. 42, no. 1, pp. 5–13, Feb. 2014. 23975584 10.1007/s15010-013-0525-9

[8] 3M. Product Clinical Data Summary: 3M Elastic Nonwoven Tape with Extended Wear Adhesive. Accessed: 5 5, 2021. [Online]. Available: https://multimedia.3m.com/mws/media/1559834O/3m-4077-clinical-summary.pdf

[9] A. C. Oliver, “Comparison of two different anti-infectious approaches after high-dose chemotherapy and autologous stem cell transplantation for hematologic malignancies in a 12-year period in British Hospital, Uruguay,” Ann. Hematol., vol. 99, no. 4, pp. 877–884, Apr. 2020. 32062742 10.1007/s00277-020-03947-1

[10] W. Owattanapanich, K. Suphadirekkul, C. Kunacheewa, P. Ungprasert, and K. Prayongratana, “Risk of febrile neutropenia among patients with multiple myeloma or lymphoma who undergo inpatient versus outpatient autologous stem cell transplantation: A systematic review and meta-analysis,” BMC Cancer, vol. 18, no. 1, p. 1126, Dec. 2018. 30445930 10.1186/s12885-018-5054-6 PMC6240267

[11] M. M. Levy, L. E. Evans, and A. Rhodes, “The surviving sepsis campaign bundle: 2018 update,” Intensive Care Med., vol. 44, no. 6, pp. 925–928, Jun. 2018. 29675566 10.1007/s00134-018-5085-0

[12] D. M. Lindberg, “The 1-hour bundle for sepsis: An update to the 2016 surviving sepsis guidelines,” New England J. Med., J. Watch Emergency Med., Massachusetts Med. Soc., Tech. Rep., 2018. [Online]. Available: https://www.jwatch.org/na46671/2018/05/10/1-hour-bundle-sepsis-update-2016-surviving-sepsis

[13] O. Kimberger, R. Thell, M. Schuh, J. Koch, D. I. Sessler, and A. Kurz, “Accuracy and precision of a novel non-invasive core thermometer,” Brit. J. Anaesthesia, vol. 103, no. 2, pp. 226–231, Aug. 2009. 10.1093/bja/aep134 19482858

[14] B. N. Jensen, “Accuracy of digital tympanic, oral, axillary, and rectal thermometers compared with standard rectal mercury thermometers,” Eur. J. Surg., vol. 166, no. 11, pp. 848–851, Oct. 2000. 11097149 10.1080/110241500447218

[15] L. Lawson, “Accuracy and precision of noninvasive temperature measurement in adult intensive care patients,” Amer. J. Crit. Care, vol. 16, no. 5, pp. 485–496, Sep. 2007. 17724246

[16] N. S. Latman, P. Hans, L. Nicholson, K. Lewis, and A. Shirey, “Evaluation of clinical thermometers for accuracy and reliability,” Biomed. Instrum. Technol., vol. 35, no. 4, pp. 259–265, 2001. 11494651

